# Assessing Discrimination and Acceptance for Lightness and Chroma During Shade Selection: A Comparison of Dental and Non-Dental Professionals

**DOI:** 10.3390/dj13040163

**Published:** 2025-04-11

**Authors:** Haruna Konishi, Yuichi Ishida, Takaharu Goto, Tetsuo Ichikawa

**Affiliations:** 1 Department of Prosthodontics & Oral Rehabilitation, Graduate School of Biomedical Sciences, Tokushima University, 3-18-15, Kuramoto, Tokushima 770-8504, Japan; konishi.haruna@tokushima-u.ac.jp (H.K.); junchan@tokushima-u.ac.jp (Y.I.); tak510@tokushima-u.ac.jp (T.G.); 2 Tokushima University, 3-18-15, Kuramoto, Tokushima 770-8504, Japan

**Keywords:** shade selection, shade guide, tooth color, lightness, chroma

## Abstract

**Background/Objectives**: Tooth color is key in determining aesthetic appearance during restorative and prosthodontic treatments. To establish a more reliable methodology for shade selection, this study investigated differences in the discrimination and acceptance of tooth color between dental and non-dental professionals, focusing on color attributes such as lightness and chroma. **Methods**: This study included 30 dentists, 30 dental technicians, 30 patients, and 30 dental students. They were asked to compare pairs of shade tabs with different lightness and chroma from the VITA Toothguide 3D-MASTER^®^ (VITA Zahnfabrik, Bad Säckingen, Germany). The number of answers in which participants could discriminate color differences (discrimination numbers) and answers in which they could not accept color differences (non-acceptance numbers) were recorded. Statistical analysis was performed using Spearman’s rank correlation coefficient, the Mann–Whitney U test, the Kruskal–Wallis test, and multiple regression analysis (*p* < 0.05). **Results**: Significant factors influencing lightness discrimination were participant group and age, while those influencing acceptance were subject group and sex. **Conclusions**: This study revealed differences in the discrimination and acceptance of lightness and chroma between dental and non-dental professionals, particularly concerning lightness. Dental technicians exhibited a higher discrimination ability and stricter acceptance of lightness and chroma, regardless of age. Based on an understanding of these characteristics, appropriate shade selection and adequate communication will be important.

## 1. Introduction

To achieve excellent esthetics in dental treatment, it is crucial to match the color of the dental prostheses and restorative materials to that of the remaining natural teeth. Even if the shape and form of dental prostheses are in harmony with those of the surrounding teeth, any discrepancy in color can lead to patient dissatisfaction. Traditionally, visual colorimetry has been the primary method for shade selection in dental practice [1], with dentists and dental technicians using dental shade guides, such as the VITAPAN Classical shade guide (VITA Zahnfabrik, Bad Säckingen, Germany), and color samples to determine the appropriate shade visually, often with patient confirmation. Reportedly, using instruments for shade selection improves the accuracy of shade matching compared to visual methods [2]. Nevertheless, it has also been reported that visual shade determination by experienced dentists is extremely important [3].

It is generally recommended that shade selection be performed by separately evaluating lightness, chroma, and hue [4,5,6,7]. The VITA Toothguide 3D-MASTER^®^ (VITA Zahnfabrik, Bad Säckingen, Germany), in contrast to the previously mentioned VITAPAN Classical, is systematically arranged according to lightness, chroma, and hue [8]. Therefore, it is designed to facilitate the selection of shade in accordance with the recommended method. Lightness refers to the brightness and translucency of the tooth color, typically described as “high lightness (bright)” or “low lightness (dark)”. Chroma refers to the intensity or saturation of the color, described as “high chroma (vivid)” or “low chroma (dull)”. However, there has been limited research on the accuracy with which clinicians perceive and adjust lightness and chroma in actual clinical practice as well as on whether the selected shade falls within an acceptable range for both patients and dental professionals.

To address these issues, it is necessary to clarify the extent to which humans can discriminate differences in tooth color as well as the level of color discrepancy deemed acceptable. Previous studies on shade selection often used dental professionals, such as dentists, dental technicians, or dental students, as test subjects. However, considering that esthetic outcomes are directly linked to patient satisfaction, it is important to clarify the acceptance of color differences between dental professionals and patients and to establish clinical guidelines based on these findings.

We evaluated two groups: dental healthcare professionals (dentists and dental technicians) and non-dental professionals (patients and dental students). Using shade tabs, we investigated how each group discriminated tooth color differences and determined the acceptable range for each color attribute, including lightness and chroma. Based on these results, we aimed to establish a more practical and reliable methodology for shade selection and contribute to the digitization of shade selection and subsequent treatment processes. This study hypothesized that there is no difference in the discrimination and acceptance of dental crown color differences, in terms of lightness and chroma, between dental professionals and non-dental professionals.

## 2. Materials and Methods

### 2.1. Participants

All participants were screened for color vision deficiency using the Ishihara Test (Ishihara’s Tests for Color Deficiency, Concise Edition, 14 Plates; Handaya, Tokyo, Japan). Participants suspected of color blindness were excluded from the study. All participants were randomly selected from the population. After the screening, this study included 120 participants, who were divided into the following four groups, each consisting of 30 subjects:

- Dentists (15 males and 15 females, aged 27–62 years, mean age: 40.6 ± 10.0 years, mean years of clinical experience: 14.3 ± 9.7)
- Dental technicians (25 males and 5 females, aged 20–70 years, mean age: 40.3 ± 14.4 years, mean years of clinical experience: 18.4 ± 13.7).
- Patients attending the University of Tokushima Dental Hospital (15 males and 15 females, aged 40–64 years, mean age: 54.0 ± 7.8 years).
- Students of the School of Dentistry, Tokushima University (15 males and 15 females, aged 21–33 years, mean age: 25.0 ± 2.6 years).

This study was conducted in accordance with the Declaration of Helsinki after obtaining approval (approval number: 4356) from the Ethics Committee of Tokushima University Hospital, and after fully explaining the experimental procedures and obtaining consent from the test subjects.

### 2.2. Shade Tab Specimens for Discrimination and Acceptance Tests

In this study, the VITA Toothguide 3D-MASTER^®^ (VITA Zahnfabrik, Bad Säckingen, Germany), which is systematically arranged according to lightness, chroma, and hue, was used. Nine shade tabs from the VITA Toothguide 3D-MASTER were used as specimens for shade selection, and the intermediate shade tab [3M2] was used as the control. For the lightness test, we prepared tabs with the same chroma and hue as the control tab but different lightness levels, and selected one lighter [2M2] and one darker tab [4M2] with the smallest lightness difference. For the chroma test, three tabs were prepared with the same lightness as the control tab but with different chroma and hue. Three tabs [3M3, 3R2.5, and 3L2.5] with higher chroma and three with lower chroma [3M1, 3R1.5, and 3L1.5] were selected from those with the smallest chroma difference.

In addition, the color difference between each shade tab was measured. The L*a*b* color space is widely recognized as a method for quantitatively representing colors. This color system, standardized by the International Commission on Illumination in 1976, is expressed in terms of L* for lightness, and a* and b* for chromaticity, which represent the hue and chroma [4,5]. In this study, the center of each shade tab was measured three times using a spectrophotometer (CM-5, Konica Minolta, Tokyo, Japan), and after calculating the average L*a*b* value, the color difference (ΔE00) from [3M2] was determined based on CIEDE 2000. The mean ΔE00 of the tabs for the lightness test was 2.3 ± 0.9, and the mean ΔE00 of the tabs for the chroma test was 2.4 ± 0.5, indicating that the color difference between the two test tabs was almost the same.

### 2.3. Discrimination and Acceptance Tests for Shade Selection

**Figure 1:**
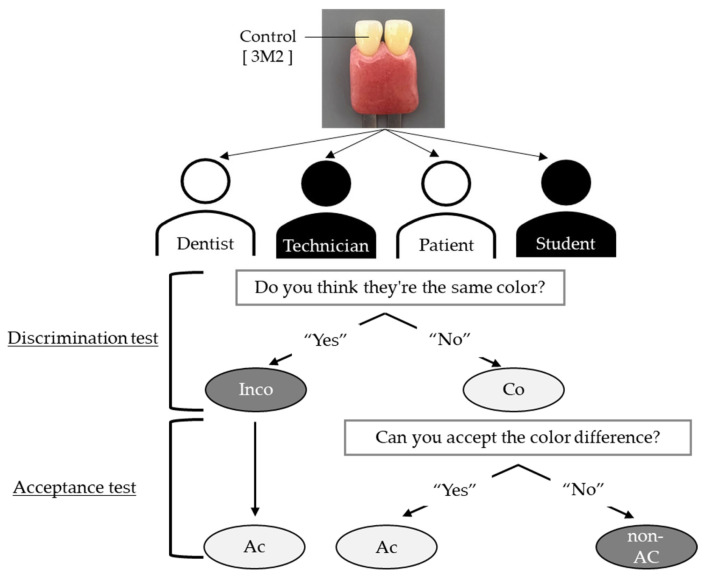
Process for the discrimination and acceptance tests (Co: correct answers, Inco: incorrect answers, Ac: acceptable, non-Ac: answers participants would not allow).

The experimental procedure is illustrated in Figure 1. Two shade tabs were fixed to a self-made gingival color holder made of auto-curing acrylic resin (PROVINICE FAST 8S, Shofu, Kyoto, Japan) and presented to the participants. One of the two shade tabs was fixed with [3M2], the central color, as a control, whereas the other was changed sequentially for presentation. Shade tabs were presented against a neutral gray background inside an observation box (Foldio3, ORANGEMONKIE, San Diego, CA, USA) that reproduced a light source at 5500 K, which is considered an ideal environment for color acquisition [9]. Based on a previous report [10], the distance from the participants’ eyes to the sample was always 30 cm. The presentations of the tabs were performed in a random order.

Regarding the discrimination test (of whether participants could recognize the color difference between the two shade tabs), participants were asked to compare each shade tab and respond to the question, “Do you think these are the same color?” If participants answered “Yes”, it was recorded as incorrect, and if they answered “No”, it was recorded as correct.

Regarding the acceptance test (of whether the color difference between the two tabs could be considered acceptable during restorative or prosthodontic treatment), if participants answered “No” in the discrimination test, they were asked: “Can you accept the shade difference?” If they answered “Yes”, it was recorded that they could accept the difference; if they answered “No”, it was recorded that they could not accept the difference. If participants answered “Yes” to the discrimination test, the color difference was automatically considered acceptable in the acceptance test.

In the lightness test, the participants were presented with two shade tabs, one with a higher lightness and one with a lower lightness than the control, each presented three times for six presentations. In the chroma test, the participants were presented with six shade tabs: three with a higher chroma and three with a lower chroma than the control, each presented once for six presentations. For each participant, the number of correct discriminations (correctly recognizing a color difference) and that of non-discriminations (failing to recognize a color difference) were recorded for the six presentations. The discrimination and acceptance rates for each presentation condition were calculated for each participant, and mean values were calculated for each participant group.

### 2.4. Statistical Analysis

The Mann–Whitney U test was used to examine the differences in discrimination and non-acceptance numbers within each participant group, differences in lightness and chroma, and differences between higher and lower levels of lightness and chroma. The Kruskal–Wallis test with Bonferroni correction was used to compare discrimination and non-acceptance rates among the four participant groups. The correlation between discrimination and non-acceptance within each group was analyzed using Spearman’s rank correlation coefficients. Multiple regression analysis was performed to investigate the factors affecting color discrimination ability and acceptance. The dependent variables were the total number of discriminations and nonacceptances. The independent variables included sex (0 = male, 1 = female), participant group (0 = dentist, 1 = dental technician, 2 = patient, 3 = student), and participant age. SPSS^®^ version 25.0 (IBM, Chicago, IL, USA) was used for all statistical analyses, with a statistical significance of 5%.

## 3. Results

Table 1 presents the results of the discrimination and acceptance tests for lightness and chroma for each group. The discrimination numbers for lightness and chroma were nearly identical between the dentist and dental technician groups, with both groups achieving discrimination rates exceeding 90%. In contrast, the patient and student groups demonstrated higher discrimination numbers for chroma than for lightness, with a significant difference between the two attributes. Furthermore, the discrimination rates for lightness in the patient and student groups were approximately 20–30% lower than those observed in the dentist and dental technician groups. Similar to discrimination, the dentist and dental technician groups showed similar acceptance numbers for lightness and chroma. However, in the patient and student groups, the non-acceptance numbers for chroma were significantly higher than those for lightness. Acceptance rates for both lightness and chroma were approximately 20% in the dental technician group and 40% in the dentist group. For chroma, the acceptance rates in the patient and student groups were similar to those in the dentist group, whereas those for lightness were higher, reaching approximately 70%.

Table 2 presents the results of the discrimination tests for each condition, that is, lightness (higher or lower than the control shade) and chroma (higher or lower than the control shade). Regarding lightness, the student group demonstrated significantly better discrimination for the lower tabs than the higher tabs. A similar trend, in which lower lightness tabs were discriminated equally well or better than higher lightness tabs, was observed in the other groups. No significant differences were found between the higher- and lower-chroma tabs in any participant group.

Table 3 presents the results of the acceptance tests for each lightness (higher or lower than the control shade) and chroma (higher or lower than the control shade). In the dentist group, the non-acceptance number for lower-lightness tabs was significantly higher than that for higher-lightness tabs. This trend was also observed in the dental technician and student groups. The nonacceptance number for higher-chroma tabs was significantly higher than that for lower-chroma tabs in the dental technician and student groups, with a similar trend observed in the other groups.

Table 4 presents the correlation between the discrimination and non-acceptance numbers in each group. A positive correlation was observed between the discrimination and non-acceptance numbers for both lightness and chroma, indicating that participants who were more capable of discriminating between colors were less likely to accept color differences. Notably, a significant positive correlation for lightness was observed in the dentist, dental technician, and patient groups, whereas a significant positive correlation for chroma was observed in the dentist group.

Table 5 summarizes the multiple regression analysis results and identifies the factors influencing discrimination and acceptance. The variance inflation factor for each independent variable ranged from 1.020 to 1.090, indicating no multicollinearity concerns. Group differences and age were identified as significant factors for lightness, with younger participants demonstrating better discrimination abilities. Sex and subject group differences were selected as significant factors for lightness, with males tending to have lower acceptance levels. For chroma, sex was the only significant factor, with males showing lower acceptance than females.

## 4. Discussion

Evaluating the color of the remaining teeth is essential in restorative and prosthetic dentistry. Many dentists and dental technicians use analog methods to assess color and then apply the color to restorations or dental prostheses. The patients then judged the applied color and its harmony by looking in the mirror. Therefore, even in the current era of rapid progress in the application of digital technology in dentistry, it is extremely important to understand how people perceive differences in tooth color. In particular, it is important to comprehend the discrimination between the two fundamental elements of “lightness” and “chroma”, which are essential for shade selection, and to assess the degree of acceptance of color differences to achieve successful esthetic dental treatment. In this study, we examined aspects of discrimination ability and acceptance from the perspectives of dental professionals, such as dentists and dental technicians, and patients. Dental students are future dental professionals, but they have not yet received sufficient education in shade selection; therefore, we selected them as test participants to include a wide range of patient groups, from young to old. Furthermore, we identified problems related to color recognition from the perspective of discrimination ability and acceptance in a manner that is relevant to clinical practice.

The discrimination ability for lightness and chroma was higher in dental professionals, such as dentists and dental technicians, than in non-dental professionals, such as patients and dental students. Additionally, the discrimination ability for lightness was significantly lower than that for chroma in non-dental professionals such as patients and dental students. In a study of dental students, Oscar et al. [11] reported that dental students, when performing visual shade matching, tended to perceive colors with small differences in chroma as matching, rather than colors with small differences in lightness. Considering that the dental students in this study had little experience in shade selection and did not receive any special training, it is thought that this group of subjects, with minimal experience in shade selection, would be less likely to notice differences in lightness than in chroma. Dental professionals, such as dentists and dental technicians, have a high level of discrimination ability for both lightness and chroma because they work in environments where precise shade selection is required in their daily clinical and technical dental work. In general, it is recommended that in shade selection, lightness should be selected first, followed by chroma [4,5,6,7]. This is because lightness determines the overall intensity of tooth color, and it has been reported that increasing the lightness of teeth, rather than the hue or chroma, is the key to success in whitening treatments [12]. However, to date, there is insufficient scientific data to support the discrimination of tooth color or acceptance of color differences. Therefore, the results of this study are expected to contribute to improvements in dental treatment.

Non-dental professionals (patients and dental students) had significantly poorer discrimination abilities than dental professionals (dentists and dental technicians) for shades of higher lightness. In addition, even for tabs with lower lightness, patients had significantly lower discrimination ability than dentists and dental technicians. Miranda [13] reported that dental students made more errors when matching light shades than when matching dark shades. Logvinenko [14] reported that for test subjects with minimal experience in shade selection, color differences meant differences in hue, and differences in lightness and chroma were not recognized as color differences. The results of these previous studies are consistent with those of the present study.

In contrast, in the lower-chroma condition, the non-dental professionals were shown to have the same discrimination ability as the dental professionals. There have been no reports examining the differences in discrimination ability and acceptance of lightness and chroma, as in this study, by comparing dental and non-dental professionals. Additionally, this study was conducted under conditions considered ideal for shade selection, and we believe that the results of this study are significant. The results of this study suggest that when the chroma of a patient’s teeth is low, there is a higher likelihood that the patient will be able to discriminate between color differences. Therefore, sufficient communication between patients, dentists, and dental technicians, as well as careful shade selection, are needed.

In addition, in the acceptance test, the dental technicians had a lower acceptance rate for differences in lightness and chroma than the other groups under all conditions. Paravina et al. [10] reported that dental technicians have higher color discrimination ability compared to dentists and students. Menini et al. [15] reported that dental technicians outperformed dentists (specialists in conservative dentistry) and implant prosthodontists in color-matching tests. Although there have been no reports to date on the acceptance of differences in lightness and chroma, the fact that dental technicians have been reported to have a higher discrimination ability than dentists suggests that they may have been more likely to perceive color differences as unacceptable, potentially influencing the results of this study.

Only a small percentage of the dentists and dental technicians judged adjacent different colors as the same and several tens of percent of the patients accepted these differences, which suggests that problems in the shade selection stage are minimal. However, even if dentists and dental technicians perceive slight color differences to be the same, some patients may not. Therefore, understanding the characteristics of color discrimination and acceptance, particularly lightness and chroma, is highly valuable in clinical practice. A significant positive correlation was observed between the discrimination and non-acceptance numbers in lightness in the patients, as well as in dental professionals, including dentists and dental technicians. Given the previously discussed differences in the discrimination ability for lightness and chroma, this suggests that patients have a relatively lower lightness discrimination ability than dental professionals. However, those who noticed the color differences tended to find it difficult to accept them. We believe that appropriate shade selection and thorough communication based on an understanding of the characteristics identified in this study are essential for the success of esthetic dental treatments.

The results of the multiple regression analysis showed that the participant groups had a significant effect on both the discrimination ability and acceptance of lightness. This is shown in Table 2 and Table 3. Regarding the effects of sex differences, previous studies have reported that females have better color discrimination than males [16,17,18], whereas others have reported the opposite [13,19,20]. Therefore, no definitive conclusion has been reached. Sex differences had a significant impact on acceptance, with males showing lower acceptance of differences in lightness and chroma. This may be attributed to the fact that the number of male dental technicians who exhibited lower acceptance was five times higher than that of female dental technicians. Age was also identified as a significant factor influencing the lightness discrimination ability, with younger participants showing a higher discrimination ability for lightness. Rods in the eyes respond to lightness [7]. According to Hathibelagal et al. [21], rod sensitivity thresholds increase rapidly from 45 years of age and older. In addition, regarding the physiological changes in visual function that occur with age, it is generally known that ocular function declines similarly to other organs and tissues, involving factors such as increased corneal thickness, decreased elasticity of the crystalline lens, and changes in transmittance [22]. This could explain the fact that the patient group with the lowest lightness discrimination ability had the highest mean age. However, it is noteworthy that the dentist and dental technician groups, which demonstrated high discrimination ability, also included participants aged 60 and over. Furthermore, multiple regression analysis showed that the β value for age was −0.190, while the β value for the participant group was −0.442, indicating that experience with shade selection had a greater impact on discrimination ability than age. This finding is particularly interesting when considering the age-related decline in visual function. The clinical significance of this study is its clarification of the differences in the discrimination and acceptance of tooth color differences between dental professionals and non-dental professionals, to establish a more practical and reliable methodology for shade selection.

One of the limitations of this study is sampling bias. Specifically, there was a significant imbalance in the male-to-female ratio in the dental technician group, which was attributable to the sampling method. To better examine the effects of sex, it is necessary to conduct a study with a balanced male-to-female ratio for each group. Additionally, the number of tabs used in the lightness and chroma tests differed in this study. Because commercially available shade tabs were used in this study, there were limitations in the types of tabs that could be used, making it difficult to prepare an equal number of tabs for both the lightness and chroma tests. In this study, we evaluated discrimination and acceptance using tooth color samples to better align with clinical conditions. The color differences ΔE00 of the shade tabs presented in this study range from 1.36 to 3.23. However, these values exceed the perceptibility threshold (0.8) and acceptability threshold (1.8) reported by Paravina [10]. Therefore, the higher discrimination rate observed in our study may be attributed to this factor. Further experiments using tooth color samples, taking previously reported thresholds [10,23] into account, are necessary.

## 5. Conclusions

Dental professionals, such as dentists and dental technicians, demonstrated high discrimination abilities for lightness and chroma, whereas non-dental professionals, such as patients and dental students, showed significantly lower discrimination abilities. Regarding the differences between lightness and chroma, non-dental professionals exhibited a significantly poorer discrimination ability than dental professionals when lightness was higher. Additionally, dental technicians exhibited a higher discrimination ability and stricter acceptance of lightness and chroma, regardless of age. Based on an understanding of these characteristics, appropriate shade selection and adequate communication are important.

## Figures and Tables

**Table 1:** Results of discrimination and acceptance tests of lightness and chroma.

Groups		Discrimination Test (Means ± SDs)			Acceptance Test (Means ± SDs)		
		Co (*n* = 30)		Discrimination Rate (%)	Non-Ac (*n* = 30)		Acceptance Rate (%)
Dentists	Lightness	5.6 ± 0.7		92.8 ± 12.1	3.2 ± 1.9		46.1 ± 31.5
	Chroma	5.5 ± 0.7		91.1 ± 12.2	3.2 ± 1.9		46.1 ± 31.8
Technicians	Lightness	5.7 ± 0.6		95.6 ± 9.7	4.4 ± 1.8		27.2 ± 30.5
	Chroma	5.9 ± 0.3		98.9 ± 4.2	4.7 ± 1.3		21.1 ± 21.0
Patients	Lightness	3.8 ± 1.8	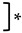	63.9 ± 30.0	1.6 ± 1.6	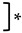	72.8 ± 27.2
	Chroma	5.3 ± 0.9		88.3 ± 15.3	3.1 ± 1.9		48.3 ± 31.1
Students	Lightness	4.5 ± 1.3	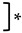	75.6 ± 21.8	1.6 ± 1.8	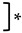	72.8 ± 29.2
	Chroma	5.4 ± 0.7		90.0 ± 12.1	3.2 ± 1.5		46.1 ± 24.6

Co: number of correct answers, non-Ac: number of answers participants would not accept, Mann–Whitney U test, * *p* < 0.05.

**Table 2:** Comparisons of discrimination rates of different lightness and chroma between each subject group.

Groups	Higher (Lightness)		Lower (Lightness)		Higher (Chroma)		Lower (Chroma)	
	Co Means ± SDs (*n* = 30)	Group Comparison	Co Means ± SDs (*n* = 30)	Group Comparison	Co Means ± SDs (*n* = 30)	Group Comparison	Co Means ± SDs (*n* = 30)	Group Comparison
Dentists	2.7 ± 0.6 (91.1 ± 21.3%)	b, c, d, e	2.8 ± 0.5 (94.4 ± 15.4%)	b, d	2.8 ± 0.5 (93.3 ± 16.1%)	d	2.7 ± 0.6 (88.9 ± 20.2%)	
Technicians	2.8 ± 0.5 (93.3 ± 16.1%)		2.9 ± 0.3 (97.8 ± 8.5%)		3.0 ± 0.0 (100.0 ± 0.0%)		2.9 ± 0.3 (97.8 ± 8.5%)	
Patients	1.9 ± 1.2 (64.4 ± 39.1%)		1.9 ± 1.2 (63.3 ± 41.4%)		2.7 ± 0.5 (90.0 ± 15.5%)		2.6 ± 0.8 (86.7 ± 27.1%)	
Students	2.0 ± 1.0 (66.7 ± 33.9%)		2.5 ± 0.7 (84.4 ± 24.3%)		2.8 ± 0.4 (94.4 ± 12.6%)		2.6 ± 0.7 (85.6 ± 24.3%)	
	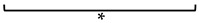							

Co: number of correct answers, numbers in parentheses indicate percentages. Statistically significant differences between groups (Kruskal–Wallis test with Bonferroni correction, *p* < 0.05) are indicated by the following letters: (a) Dentists vs. Technicians, (b) Dentists vs. Patients, (c) Dentists vs. Students, (d) Technicians vs. Patients, (e) Technicians vs. Students, and (f) Patients vs. Students. Within-group differences (Mann–Whitney U test, * *p* < 0.05) are indicated by asterisks.

**Table 3:** Comparisons of acceptance rates of different lightness and chroma between each subject group.

Groups	Higher (Lightness)		Lower (Lightness)		Higher (Chroma)		Lower (Chroma)	
	Non-Ac Means ± SDs (*n* = 30)	Group Comparison	Non-Ac Means ± SDs (*n* = 30)	Group Comparison	Non-Ac Means ± SDs (*n* = 30)	Group Comparison	Non-Ac Means ± SDs (*n* = 30)	Group Comparison
Dentists	1.1 ± 1.2 (62.2 ± 38.9%)	a, d, e	2.1 ± 1.2 (30.0 ± 39.5%)	b, c, d, e	1.9 ± 1.0 (37.8 ± 34.7%)	a, d, e	1.4 ± 1.1 (54.4 ± 37.6%)	e
	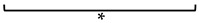							
Technicians	2.3 ± 0.9 (24.4 ± 31.5%)		2.1 ± 1.2 (30.0 ± 40.4%)		2.6 ± 0.7 (12.2 ± 23.9%)		2.1 ± 1.0 (30.0 ± 34.3%)	
					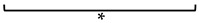			
Patients	0.7 ± 1.1 (77.8 ± 35.4%)		1.0 ± 1.2 (67.8 ± 40.6%)		1.6 ± 1.1 (45.6 ± 37.6%)		1.5 ± 1.1 (51.1 ± 36.9%)	
Students	0.6 ± 1.0 (81.1 ± 32.4%)		1.1 ± 1.2 (64.4 ± 40.1%)		2.0 ± 0.9 (34.4 ± 30.9%)		1.3 ± 0.9 (57.8 ± 31.5%)	
					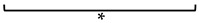			

Non-Ac: number of answers participants would not accept, numbers in parentheses indicate percentages. Statistically significant differences between groups (Kruskal–Wallis test with Bonferroni correction, *p* < 0.05) are indicated by the following letters: (a) Dentists vs. Technicians, (b) Dentists vs. Patients, (c) Dentists vs. Students, (d) Technicians vs. Patients, (e) Technicians vs. Students, and (f) Patients vs. Students. Within-group differences (Mann–Whitney U test, * *p* < 0.05) are indicated by asterisks.

**Table 4:** Correlation analysis between the number of discriminations and non-acceptances.

	Dentists		Technicians		Patients		Students	
	R	*p*-Value	R	*p*-Value	R	*p*-Value	R	*p*-Value
Lightness	0.500 *	*p* < 0.01	0.490 *	*p* < 0.01	0.603 *	*p* < 0.01	0.285	0.127
Chroma	0.481 *	*p* < 0.01	0.178	0.348	0.314	0.091	0.09	0.637

Spearman’s rank correlation coefficient, * *p* <0.05.

**Table 5:** Results of multiple regression analysis (*n* = 120).

Variable	Discrimination				Acceptance				VIF
	Lightness		Chroma		Lightness		Chroma		
	β	*p*-Value	β	*p*-Value	β	*p*-Value	β	*p*-Value	
Sex	−0.037	0.659	−0.033	0.723	−0.213	0.012 *	−0.211	0.022 *	1.02
Age	−0.19	0.032 *	−0.119	0.213	−0.016	0.849	−0.074	0.436	1.09
Subject group	−0.442	<0.01 *	−0.157	0.101	−0.39	<0.01 *	−0.108	0.253	1.077

β: standardized partial regression coefficient, VIF: variance inflation factor, * *p* < 0.05.

## Data Availability

Data are contained within the article.

## References

[1] Hardan L., Bourgi R., Cuevas-Suárez C.E., Lukomska-Szymanska M., Monjarás-ávila A.J., Zarow M., Jakubowicz N., Jorquera G., Ashi T., Mancino D. (2022). Novel Trends in Dental Color Match Using Different Shade Selection Methods: A Systematic Review and Meta-Analysis. Materials.

[2] Morsy N., Holiel A.A. (2023). Color Difference for Shade Determination with Visual and Instrumental Methods: A Systematic Review and Meta-Analysis. Syst. Rev..

[3] Abu-Hossin S., Onbasi Y., Berger L., Troll F., Adler W., Wichmann M., Matta R.-E. (2023). Comparison of Digital and Visual Tooth Shade Selection. Clin. Exp. Dent. Res..

[4] Jouhar R., Ahmed M.A., Khurshid Z. (2022). An Overview of Shade Selection in Clinical Dentistry. Appl. Sci..

[5] Mohammed A.O., Mohammed G.S., Mathew M., Alzarea B., Bandela V. (2022). Shade Selection in Esthetic Dentistry: A Review. Cureus.

[6] Basavanna R., Gohil C., Shivanna V. (2013). Shade Selection. Int. J. Oral Health Sci..

[7] Fondriest J. (2003). Shade Matching in Restorative Dentistry: The Science and Strategies. Int. J. Periodontics Restor. Dent..

[8] Gómez-Polo C., Fraile J.F., López N.Q., Muñoz M.P., Lobato M., Montero J. (2024). Three-Dimensional Representation of the Vita Toothguide 3D-Master: An in Vivo Clinical Study. J. Esthet. Restor. Dent..

[9] Wee A.G., Meyer A., Wu W., Wichman C.S. (2016). Lighting Conditions Used during Visual Shade Matching in Private Dental Offices. J. Prosthet. Dent..

[10] Paravina R.D., Ghinea R., Herrera L.J., Bona A.D., Igiel C., Linninger M., Sakai M., Takahashi H., Tashkandi E., Del Mar Perez M. (2015). Color Difference Thresholds in Dentistry. J. Esthet. Restor. Dent..

[11] Pecho O.E., Pérez M.M., Ghinea R., Della Bona A. (2016). Lightness, Chroma and Hue Differences on Visual Shade Matching. Dent. Mater..

[12] Carey C.M. (2014). Tooth Whitening: What We Now Know. J. Evid. Based Dent. Pract..

[13] Miranda M.E. (2012). Effect of Gender, Experience, and Value on Color Perception. Oper. Dent..

[14] Logvinenko A.D. (2015). The Geometric Structure of Color. J. Vis..

[15] Menini M., Rivolta L., Manauta J., Nuvina M., Kovacs-Vajna Z.M., Pesce P. (2024). Dental Color-Matching Ability: Comparison between Visual Determination and Technology. Dent. J..

[16] Pecho O.E., Ghinea R., Perez M.M., Della Bona A. (2017). Influence of Gender on Visual Shade Matching in Dentistry. J. Esthet. Restor. Dent..

[17] Gasparik C., Grecu A.G., Culic B., Badea M.E., Dudea D. (2015). Shade-Matching Performance Using a New Light-Correcting Device. J. Esthet. Restor. Dent..

[18] Haddad H.J., Jakstat H.A., Arnetzl G., Borbely J., Vichi A., Dumfahrt H., Renault P., Corcodel N., Pohlen B., Marada G. (2009). Does Gender and Experience Influence Shade Matching Quality?. J. Dent..

[19] Makhloota M., Köroğlu A., Turhan Bal B., Velioğlu N. (2024). Comparison between Direct and Indirect “Digital Image” Dental Visual Shade Matching Considering the Effect of Clinical Experience and Gender. J. Esthet. Restor. Dent..

[20] Paravina R.D. (2002). Evaluation of a Newly Developed Visual Shade-Matching Apparatus. Int. J. Prosthodont..

[21] Hathibelagal A.R., Bharadwaj S.R., Yadav A.R., Subramanian A., Sadler J.R.E., Barbur J.L. (2020). Age-Related Change in Flicker Thresholds with Rod- A Nd Cone-Enhanced Stimuli. PLoS ONE.

[22] Maitre J., Gasnier Y., Bru N., Jully J.L., Paillard T. (2013). Discrepancy in the Involution of the Different Neural Loops with Age. Eur. J. Appl. Physiol..

[23] Douglas R.D., Steinhauer T.J., Wee A.G. (2007). Intraoral Determination of the Tolerance of Dentists for Perceptibility and Acceptability of Shade Mismatch. J. Prosthet. Dent..

